# Quantification of the effects of architectural traits on dry mass production and light interception of tomato canopy under different temperature regimes using a dynamic functional–structural plant model

**DOI:** 10.1093/jxb/eru356

**Published:** 2014-09-02

**Authors:** Tsu-Wei Chen, Thi My Nguyet Nguyen, Katrin Kahlen, Hartmut Stützel

**Affiliations:** ^1^Institute of Horticultural Production Systems, Leibniz Universität Hannover, Herrenhäuser Strałe 2, D-30419 Hannover, Germany; ^2^Department of Vegetable Crops, Geisenheim University, Von-Lade-Straße 1, D-65366 Geisenheim, Germany

**Keywords:** Canopy photosynthesis, dynamic model, functional–structural plant model, light interception, plant architecture, temperature, tomato.

## Abstract

There is increasing interest in evaluating the environmental effects on crop architectural traits and yield improvement. However, crop models describing the dynamic changes in canopy structure with environmental conditions and the complex interactions between canopy structure, light interception, and dry mass production are only gradually emerging. Using tomato (*Solanum lycopersicum* L.) as a model crop, a dynamic functional–structural plant model (FSPM) was constructed, parameterized, and evaluated to analyse the effects of temperature on architectural traits, which strongly influence canopy light interception and shoot dry mass. The FSPM predicted the organ growth, organ size, and shoot dry mass over time with high accuracy (>85%). Analyses of this FSPM showed that, in comparison with the reference canopy, shoot dry mass may be affected by leaf angle by as much as 20%, leaf curvature by up to 7%, the leaf length:width ratio by up to 5%, internode length by up to 9%, and curvature ratios and leaf arrangement by up to 6%. Tomato canopies at low temperature had higher canopy density and were more clumped due to higher leaf area and shorter internodes. Interestingly, dry mass production and light interception of the clumped canopy were more sensitive to changes in architectural traits. The complex interactions between architectural traits, canopy light interception, dry mass production, and environmental conditions can be studied by the dynamic FSPM, which may serve as a tool for designing a canopy structure which is ‘ideal’ in a given environment.

## Summary

A dynamic function–structural plant model of tomato is proposed and evaluated under two temperature regimes. The model reveals that architectural traits may affect shoot dry mass by up to 20%.

## Introduction

Increasing crop productivity is an important objective of current plant science. Many approaches, such as improving photosynthesis (Zhu *et al*., 2010, 2012; Evans, 2013), nutrient use efficiency (Xu *et al.*, 2012), and tolerance to biotic and abiotic stress (Munns and Tester, 2008; Roy *et al.*, 2011), have been proposed in the past decades. An interesting and important question is to what extent alterations in single processes and traits may improve yield at the canopy level (Zhu *et al.*, 2012; Evans, 2013). Without credible assessment of these impacts ‘prioritizing the choice of target is a gamble’ (Evans, 2013). To assess the impact of a single trait on improving yield, in recent years plant scientists and statisticians have started to develop tools and methods to evaluate the relative importance of these targets.

Although there are urgent needs for and increasing interest in using crop models to quantify the relative importance of plant traits in improving yields, reliable crop models are not available (Evans, 2013). A big challenge is the prediction of canopy photosynthesis in fluctuating environments. Most of the existing models predicting canopy photosynthesis consist of three main components: whole-plant leaf area (or leaf area index, LAI), light interception by leaves, and photosynthetic rates of leaves. Accurate prediction of leaf area under a certain range of environmental conditions remains a challenge. One reason is that leaf area is strongly affected by many factors such as temperature, vapour pressure deficit (VPD), and environmental stress (Tardieu *et al.*, 2000; Heuvelink and Dorias, 2005; Najla *et al.*, 2009). Furthermore, changes in environmental conditions at the leaf level may not necessarily influence final plant leaf area (Granier and Tardieu, 2009), since the latter is a function not only of individual leaf area but also of the number of leaves and leaf senescence (Yin *et al.*, 2000).

Significant temperature effects have been shown to occur on the rates of tissue initiation and expansion, and the duration of tissue expansion (Tardieu *et al.*, 2000; van der Ploeg and Heuvelink, 2005; Granier and Tardieu, 2009; Parent and Tardieu, 2012). This indicates that temperature has strong influences on architectural traits such as the leaf number, leaf area, and internode length. These modifications of leaf and stem properties by temperature alter the canopy structure such as crown density and leaf dispersion, consequently affecting light interception and dry mass production. However, there are only a very limited number of studies on quantifying the influence of temperature regime on plant architecture and light regime at the canopy level.

Knowledge of the amount of light intercepted by individual leaves or layers in the canopy is required to calculate the rate of photosynthesis. One classical approach is using Beer–Lambert’s law, according to which light passing through the canopy is reduced exponentially with LAI and a light extinction coefficient, *k* (Monsi and Saeki, 1953; Hirose, 2005). However, *k* is not a constant in a growing canopy as it varies with developmental stage, plant structure, canopy configurations (Evers *et al.*, 2009), and architectural traits, such as leaf shape, leaf angle, and internode length (Hirose, 2005; Kahlen *et al.*, 2008). The importance of architectural traits for light interception has been widely reported (Takenaka, 1994; Zhu *et al*., 2010, 2012; Sarlikioti *et al.*, 2011; Song *et al.*, 2013). For example, plants with longer internodes increase light harvest (Weijschede *et al.*, 2008). Leaf curvature (curvature of the midrib) has received some attention mostly in maize (e.g. Espana *et al.*, 1999; Ford *et al.*, 2008). Leaf angle has been considered as an important architectural trait for a very long time (Evers *et al.*, 2009; Sarlikioti *et al.*, 2011; Song *et al.*, 2013). Furthermore, canopy structure and these architectural traits change dynamically with the growth of plants. The complexity of architectural influence on light interception establishes the need to combine all architectural information in a functional–structural model (FSPM) to describe canopy architecture more accurately, which in turn determines light interception and canopy photosynthesis (Vos *et al.*, 2010; Buck-Sorlin *et al.*, 2011; Song *et al.*, 2013). The influence of architectural traits on canopy photosynthesis has been evaluated by static FSPMs in tomato (Sarlikioti *et al.*, 2011) and in rice (Song *et al.*, 2013). However, these effects were analysed based on static canopy architecture at a specific developmental stage of the plant. The long-term impacts of architectural changes on canopy photosynthesis in a dynamic environment remain unknown.

Lindenmayer systems (L-systems) are a widely used approach to construct dynamic plant architectural models using empirically derived functions. L-systems were first used to describe the development of multicellular organisms (Lindenmayer, 1968). They have been extended to plant growth modelling for many crops such as rose (Buck-Sorlin *et al.*, 2011), kiwi (Cieslak *et al.*, 2011), wheat (Evers *et al.*, 2009), cucumber (Kahlen *et al.*, 2008; Kahlen and Stützel, 2011; Wiechers *et al.*, 2011), and tomato (Najla *et al.*, 2009). L-systems have been widely used because they are an elegant formalism for generating branching structures and describing complicated structural dynamics (Prusinkiewicz and Lindenmayer, 1990). Virtual plants expressed by L-systems interfacing with a light environmental model allow estimation of the distribution of irradiance from direct and indirect light sources at the leaf level (e.g. Cieslak *et al.*, 2008).

The objective of this study was to assess the potential impacts of architectural traits on canopy light interception and canopy photosynthesis under different temperature regimes. Using tomato (*Solanum lycopersicum* L.) as a model crop, this objective was achieved in four steps. (i) Experiments were conducted for parameterizing models for single organ expansion and shape alteration. (ii) Combining these models at the organ level with an L-system and light model, a dynamic FSPM for tomato canopies was constructed. (iii) The model was evaluated at both the single organ and plant level using an independent data set. (iv) The FSPM was used to quantify the effects of architectural traits on canopy dry mass production.

## Materials and methods

### Plant cultivation and data collection

Tomato (*S. lycopersicum* L. ‘Pannovy’, Syngenta) was used in all experiments. Five experiments were conducted in the growth chambers and greenhouses of Leibniz Universität Hannover, Germany. Experiments 1, 2, and 3 were carried out in growth chambers with a variation of air temperatures, VPDs, and light intensities (Supplementary Table S1 available at *JXB* online) to obtain data for parameterization of the leaf model. Experiments 4 and 5 were performed in greenhouses in 2009 and 2010, respectively, for parameterization (experiment 4) and evaluation (experiment 5) of the canopy model. The plants in all experiments were raised in the same way throughout, starting with sowing into small rock wool cubes of 2.5 cm×2.5 cm×2.5cm in growth chambers with a light intensity of 300 μmol m^–2^ s^–1^ photosynthetically active radiation (PAR), VPD of 0.8 kPa, and day/night temperatures of 22/18 °C. The seedlings at the cotyledon stage were transplanted to larger rock wool cubes of 10 cm×10 cm×10cm. When the first true leaves appeared, seedlings were transplanted into the hydroponic system with the desired treatments for growth chamber experiments. For the greenhouse experiments, the plants were maintained in the growth chambers until they had five true leaves before transplanting to the greenhouses (for details, see Supplementary Table S2). In all experiments, side shoots were removed daily to maintain one stem per plant, which was trained upright. Plant protection was conducted as necessary to keep plants free from damage.

In the growth chamber experiments, each treatment consisted of six plants grown in three 50 litre hydroponic containers. The nutrient solution had an electrical conductivity (EC) of 2 dS m^–1^ with concentrations of 175mg l^–1^ N, 40mg l^–1^ P, 300mg l^–1^ K, 40mg l^–1^ Mg, 175mg l^–1^ Ca, 120mg l^–1^ S, and 0.8mg l^–1^ Fe. Styrofoam covered the surface of the nutrient solution to prevent the growth of algae and to maintain the plants floating on the nutrient solution. Holes in the Styrofoam of the size of the rock wool cubes fixed the plants inside these holes. The nutrient solution was renewed weekly. Air stones supplied air to the nutrient solution.

In experiment 4, greenhouse ventilators were opened when the day temperature reached 24 °C. The nutrient solution was the same as in the growth chamber experiments except that a drip irrigation system was used. The tomatoes were planted on rock wool slabs (Grodan B.V, Roermond, The Netherlands) with 1 m spacing between rows and within each row. There were four replications each consisting of four plants. Experiment 5 was established in two greenhouses with 22/18 °C (low) and 32/28 °C (high) day/night temperature. Ventilators opened when the day temperature reached 24 °C and 34 °C in the low and high temperature regimes, respectively. The drip irrigation system was similar to that of experiment 4. There were two replications, each consisting of eight plants.

**Fig. 1.**
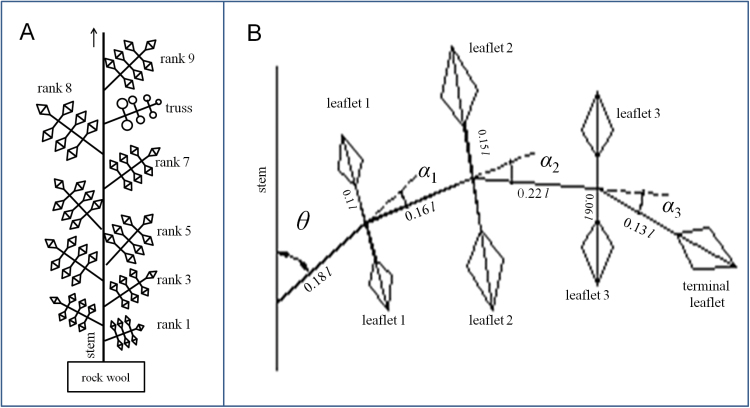
Representation of the architecture of a tomato plant (A) and that of a leaf with leaf length=*l* (B). Leaf angle (θ) is the angle between stem and petiole. Leaf curvature is defined as the sum of α_1_, α_2_, and α_3_. Reference ratio of α_1_:α_2_:α_3_=1:2:2. Reference area of leaflet 1:leaflet 2:leaflet 3:terminal leaflet=0.12:0.17:0.13:0.16. Reference ratio of the length and width of all leaflets is 1.33. Reference values were derived from leaves grown in experiment 1.

In experiments 1 and 2, the lengths of the leaves at rank 8 (i.e. the leaves below the first trusses; Fig. 1A) were measured daily, and the lengths of the other leaves were measured weekly. All the lengths were measured manually using a ruler. Leaf length was defined as the distance from the tip of the terminal leaflet to the insertion of the rachis on the stem. At the end of these experiments, the area of each leaf was measured using a LI-COR 3100 area meter (LI-COR, Lincoln, NE, USA) to establish the relationship between leaf length and area. In addition, a leaf was counted when its length was ≥1cm, and the number of leaves was counted daily. Internode lengths at rank 8 were measured daily in experiments 1, 2, and 3.

In experiment 4, leaf angle, leaf curvature, and leaf senescence were recorded. Leaf angle is the angle between the stem and the line between leaf insertion and the point where the first leaflet appeared, while leaf curvature is the sum of the angles describing the curvature of the midrib (Fig. 1B). The points to calculate these angles were obtained from digitizing using a Fastrak 3D digitizer (Polhemus Inc., Colchester, VT, USA). A leaf was defined as senescent when > 30% of its lamina area (by visual assessment) had turned yellow. In addition, leaf optical properties (reflectance and transmittance) of the full spectrum of the upper and the lower sides of leaves were also measured for different leaf ages: young, mature, and old leaves of three plants per treatment using a LI-1800 spectrometer (Li-Cor).

In experiment 5, lengths of leaves and internodes at ranks 8 and 13, the number of leaves, and plant height were recorded twice a week. The first truss appeared after the leaf at rank 8, and truss clusters alternated with every three leaves throughout. Therefore, the leaf appearance rate of leaves above rank 8 was three times the truss appearance rate on average. The leaf appearance rate was calculated as the slope of the relationship between time and leaf number. Shoot dry weight and plant leaf area were sampled once a week starting 28 d after the first true leaf appeared (DAFLA). Air temperature, VPD, and PAR in the greenhouse experiments were recorded hourly. At the end of all experiments, all plant organs were dried at 70 °C for at least 96h and weighed to determine dry mass.

Additionally, to enhance the data set for deriving base temperature, data for maximum leaf elongation rate of the leaves at rank 5 from Fanwoua (2007) were used. In this work (referred to as experiment 6), tomato cv. Pannovy was grown in growth chambers under different temperature regimes: 8/12, 14/18, 20/24, 26/30, and 32/36 °C for day/night temperature.

### Canopy composition and light model

An L-system was used to construct the model for plant architecture. The model was established using *lpfg* where L-system-specific constructs were added to the C++ programming language (Karwowski and Lane, 2008). A Quasi-Monte Carlo algorithm-based light model was utilized for estimating light absorption of the canopy (for details, see Kahlen and Stützel, 2011).

The virtual canopy comprised 16 plants (4×4), in which four plants in the centre of the canopy were analysed using mean values. The virtual ground was covered by a white rectangle, reflecting 80% of incident light without transmittance, which is in agreement with the set-up of the greenhouse experiments.

### Geometrical properties of leaf

The arrangement of leaves at the main stem was defined by a phyllotaxis angle of 144 ° (Najla *et al.*, 2009). Each leaf consisted of seven leaflets, one terminal leaflet and three pairs of lateral leaflets arranged opposite to each other (Fig. 1B). In the L-systems, each leaflet was represented by a rhombus. Based on data from experiment 1, geometrical relationships between leaflet length, leaflet width, petiole length of leaflets, and leaf length were derived. Petioles and internodes were interpreted by cylinders.

### Architecture model of leaf and internode

The elongation rate *E*_l_(*t*, *TS*, *r*) (cm d^–1^) of a leaf at time *t* (d), with a given temperature sum, *TS* (°Cd, calculated by accumulating the difference between the average air temperature and the base temperature each day from the date of leaf appearance) and at rank *r* was calculated as the product of the maximum leaf elongation rate of the leaf at rank 8, *E*_l,max_(*t*) (cm d^–1^), normalized effect of temperature sum, *E*_l,norm_(*TS*), and normalized rank effect, *R*_l,norm_(*r*):

$$E_{\text{l}}\left(t,TS,r\right)=E_{\text{l},\text{max}}\left(t\right)\times E_{\text{l},\text{norm}}\left(TS\right)\times R_{\text{l},\text{norm}}\left(r\right)$$ (1)

*E*_l,max_(*t*) was computed based on the approach proposed by Tardieu *et al.* (2000), but only depending on daily temperature and VPD. Additionally, the model assumed that when temperature is above an optimal temperature, *T*_opt_ (°C), then *E*_l,max_(*t*) would decrease at the same rate as it increases in the range of temperatures below the *T*_opt_:

$$\begin{array}{l} E_{\text{l},\text{max}}\left(t\right)=\left[T\left(t\right)–T_{\text{b}}\right]\;\left[\begin{array}{l} a_{\text{El},\text{max}}+b_{\text{El},\text{max}}\\ \times \text{VPD}\left(t\right) \end{array}\right]\;\\ \text{for }T_{\text{b}}\leq T\left(t\right)\;\leq T_{\text{opt}} \end{array}$$ (2a)

$$\begin{array}{l} E_{\text{l},\text{max}}\left(t\right)=\left[\text{2}T_{\text{opt}}–T\left(t\right)–T_{\text{b}}\right]\;\left[\begin{array}{l} a_{\text{El},\text{max}}+b_{\text{El}.\text{max}}\\ \times \text{VPD}\left(t\right) \end{array}\right]\;\\ \text{for }T\left(t\right)\;>T_{\text{opt}} \end{array}$$ (2b)

where *T*(*t*) and *T*_b_ are air temperature at time *t* and base temperature (°C), respectively. VPD(*t*) is the VPD (kPa) at time *t*. The base temperature of 6.8 °C was obtained by extrapolation of a linear relationship between normalized maximum leaf elongation rates and air temperature. The normalized effect of temperature sum, *E*_l,norm_(*TS*), was considered as a bell-shaped function depending on leaf temperature sum:

$$E_{\text{l},\text{norm}}\left(TS\right)=\text{exp}\left\{–0.\text{5}{\left[\left(TS–TS_{\text{l},\text{max}}\right)/h_{\text{l}}\right]}^{\text{2}}\right\}$$ (3)

where *TS*_l,max_ (°Cd) is the temperature sum required by a leaf to reach its maximum elongation rate. Normalized rank effects, *R*_r,norm_(*r*), on leaf elongation were assumed to follow a bell-shaped function for ranks below 14:

$$R_{\text{r},\text{norm}}\left(r\right)=\text{exp}\{–0.\text{5}{\left[\left(r–R_{\text{max}}\right)/h\right]}^{\text{2}}\}$$ (4)

where *R*_max_ is the rank where a leaf has the maximum leaf length. Due to the short duration of the growth chamber experiments, the measurement of rank effects could be done only for leaves on ranks 1–13. The maximum elongation rate *E*_l,max_ of leaves above rank 13 was assumed to be the same as that of the leaves at rank 13.

Leaf length *L*_l_ (cm) was calculated as the cumulative *E*_l_, and the area of a leaf, *A*_l_ (cm^2^), was computed based on the relationship between leaf length and area for this specific cultivar:

$$A_{\text{l}}=a_{\text{Al}}\times L_{\text{l}}{}^{\text{g}}$$ (5)

where *a*_Al_ and *g* are empirical coefficients. The leaf appearance rate at time *t*, *R*_l_(*t*) (leaf d^–1^), was:

$$R_{\text{l}}\left(t\right)=a_{\text{r}}\times \text{ln}\left[T\left(t\right)\right]–b_{\text{r}}\text{ for }T\left(t\right)\;\leq \text{3}0\;{}^{\circ}\text{C}$$ (6a)

$$R_{\text{l}}\left(t\right)=R_{l}{}_{\text{max}}\text{ for }T\left(t\right)>\text{3}0\;{}^{\circ}\text{C}$$ (6b)

where *a*_rl_ and *b*_rl_ are empirical parameters and, if *R*_l_(*t*) reached its maximum value, *R*_lmax_, at 30 °C, a further increase in temperature did not increase *R*_l_(*t*). The number of leaves was calculated as the integral of *R*_l_ over time. Total plant leaf area, *A*_p_ (cm^2^ plant^–1^), was the accumulated leaf area of all leaves on the main stem.

Leaf angle, θ (°), and leaf curvature, *C*_l_ (°), were assumed to be leaf length dependent and followed logistic and linear functions for leaf angle and curvature, respectively.

$$\theta ={\text{a}}_{\theta }\times \left\{\text{1}–\text{exp}\left[–\left(b_{\theta }\times L_{\text{l}}\right)\right]\right\}$$ (7)

$$C_{\text{l}}=a_{\text{Cl}}–b_{\text{Cl}}\times L_{\text{l}}\text{ for }L_{\text{l}}\leq \text{5}0\text{ cm}$$ (8b)

$$C_{\text{l}}=a_{\text{1Cl}}+b_{\text{1Cl}}\times L_{\text{l}}\text{ for }L_{\text{l}}>\text{ 5}0\text{ cm }$$ (8b)

where *a*_θ_, *b*_θ_, *a*_Cl_, and *b*_Cl_ are empirical coefficients.

Internode elongation rate, *E*_i_ (cm d^–1^), was modelled as the product of the maximum internode elongation rate, *E*_i,max_(*t*) (cm d^–1^), and normalized internode elongation rate, *E*_i,norm_. *E*_i,max_ was computed similarly to *E*_l,max_ but was considered to be dependent on temperature and PAR:

$$E_{\text{i},\text{max}}\left(t\right)=\left[T\left(t\right)–T_{\text{bi}}\right]\;\left[a_{\text{Ei},\text{max}}–b_{\text{Ei},\text{max}}\times \text{PAR}\left(t\right)\right]$$ (9)

$$E_{\text{i},\text{norm}}\left(TS\right)=\text{exp }\left\{–0.\text{5}{\left[\left(TS–TS_{\text{i},\text{max},}\right)/h_{\text{i}}\right]}^{\text{2}}\right\}$$ (10)

where *T*_bi_ is the base temperature for internode growth. It was derived using the same procedure as base temperature for leaf growth. *TS* is temperature sum (°Cd) and *TS*_i,max_ is the temperature sum when the internode reaches its maximum elongation rate (°Cd). PAR(*t*) is photosynthetically active radiation of day *t* (μmol m^–2^ s^–1^). The parameters *h*_i_, *a*_Ei,max_, and *b*_Ei,max_ are shape coefficients. Internode length, *L*_i_ (cm), is the accumulation of *E*_i_. Internode diameter, *D*_i_ (cm), increases linearly with the age of the internode in terms of *TS*:

$$D_{i}=a_{\text{Di}}+b_{\text{Di}}\times TS$$ (11)

where *a*_Di_ and *b*_Di_ are empirical parameters.

### Dry matter production

Dry matter production of a leaf, *W*_l_ (g d^–1^), was the product of leaf area (*A*_l_, m^2^), light absorption (*I*_abs,_ J m^–2^ d^–1^), and light use efficiency, ε (g CO_2_ J^–1^):

$$W_{\text{l}}=I_{\text{abs}}\times \varepsilon \left(I_{\text{abs}}\right)\times \{\text{1}–\kappa {\left[T\left(t\right)–T*\right]}^{\text{2}}\}\times A_{\text{l}}$$ (12)

where ε(*I*_abs_) is an empirical light-dependent function for tomato derived from the literature (Warren-Wilson *et al.*, 1992), and the term {1–κ[*T*(*t*)–T*]^2^}, a concave parabola reaching its maximum at T*, describes the temperature response of light use efficiency. Parameter κ (0.0013 °C^–2^) and T* (25 °C) for tomato were taken from Gent and Seginer (2012). To simulate leaf senescence when the temperature sum of a leaf is larger than a threshold value, *TS*_l,sen_(°Cd), representing that 30% visible yellow symptoms can be observed on leaf lamina, this leaf no longer produces dry mass.

Plant dry weight, *W*_p_(g), is then the accumulation of dry weight produced by all leaves. Thus, shoot dry weight, *W*_s_(g), is a proportion of plant dry weight:

$$W_{\text{s}}=\mu \times W_{\text{p}}$$ (13)

where μ is a partitioning factor of dry weight to above-ground organs.

### Simulation procedures and model evaluation

Simulations were run for two different temperature regimes with the measured climate data in experiment 5 (set point temperatures 22/18 °C, referred to as ‘LT’, and 32/28 °C, referred to as ‘HT’). Simulations were run five times with randomized changes of phyllotaxis angle (144±10 °). At the organ level, measured and simulated leaf and internode lengths over time were compared for rank 8 and 13. At the canopy level, measured and simulated leaf number, plant height (sum of all internode lengths of a plant), leaf area, and shoot dry weight were evaluated. Measured total leaf area and shoot dry weight were compared with the simulated data, which are the average values of the four plants in the middle of the virtual canopy. Statistics of comparison were root mean square deviation (RMSD), bias, and accuracy (%):

$$\text{RMSD}=\sqrt{\frac{1}{\text{n}}\overset{\text{n}}{\underset{\text{i}=1}{\sum }}{\left(x_{\text{i}}-y_{\text{i}}\right)}^{2}}$$ (14a)

$$\text{Bias}=\frac{1}{\text{n}}\overset{\text{n}}{\underset{\text{i}=1}{\sum }}(x_{\text{i}}–y_{\text{i}})$$ (14b)

$$\text{Accuracy}=1–\frac{\text{RMSD}}{\frac{1}{\text{n}}\sum_{\text{i}=1}^{\text{n}}(x_{\text{i}})}$$ (14c)

where *x*_i_ and *y*_i_ are measured and simulated data, respectively (Kobyashi and Salam, 2000; Kahlen and Stützel, 2011).

### Analyses of morphological traits

To quantify the effect of the changes in morphological traits on light interception and dry mass production, analyses were conducted separately for both temperature regimes. Leaf angles (θ), leaf curvature angles (α_1_+α_2_+α_3_), and internode lengths were simulated with 70–130% of the reference values (100%). Furthermore, the ratio of curvature angles, α_1_:α_2_:α_3_, was modified (reference=1:2:2; MC1 scenario=1:1:1; MC2 scenario=1:1:2; MC3 scenario=1:2:3; MC4 scenario=2:1:1). The leaflet length/width ratio was changed between 0.5 and 1.5 (reference=1.33). Different arrangements of the leaflets, which was represented by the variation in area ratio between leaflets, were simulated (reference area ratio of leaflet 1:leaflet 2:leaflet 3:terminal leaflet=0.12:0.17:0.13:0.16; ML1=0.143:0.143:0.143:0.142; ML2=0.2:0.15:0.11:0.08; ML3=0.08:0.15:0.17:0.2). When one morphological trait was changed, all the other traits were kept identical to those of the reference plants.

### Canopy light interception

Four virtual sensors of 1 m^2^ were added to the ground in the middle of the central plants to estimate light transmittance through the canopy (*Q*_t_, μmol m^–2^ s^–1^). The sensors had no reflectance and no transmittance. The estimated absorption therefore corresponded to the transmittance of light through the canopy. To avoid effects of the virtual sensors on light distribution, they were only available on 28, 44, 56, and 64 DAFLA. The light extinction coefficient (*k*) was calculated by Beer–Lambert’s law (Monsi and Saeki, 1953)

$$Q_{\text{t}}/Q_{0}=e^{–k\cdot \text{LAI}}$$ (15)

where LAI is the leaf area index (m^2^ leaf area per m^2^ ground area) and *Q*_0_ (μmol m^–2^ s^–1^) is the total incoming irradiance.

## Results

The climate data in experiment 5 are shown in Supplementary Fig. S1 at *JXB* online. The values of model parameters are summarized in Supplementary Table S3. Details for parameterization can be found in Supplementary Figs S2–S6. The adaxial and abaxial sides of tomato leaves reflect 7.3% and 12.7% of incident light and transmit 2.4% and 2.5% of incident light, respectively.

### Model evaluation at the organ level

**Fig. 2.**
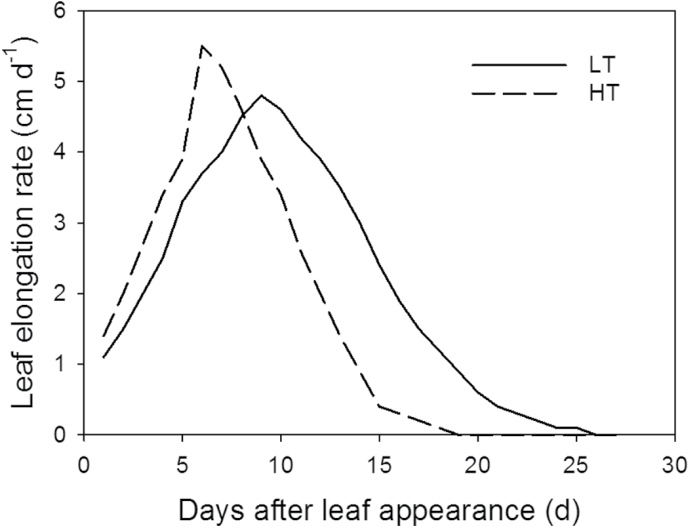
Time course of simulated leaf elongation rates of the leaves at rank 8. Solid and dashed lines represent the simulated leaf elongation rates at 22/18 °C (LT) and 32/28 °C (HT) day/night temperature conditions.

**Fig. 3.**
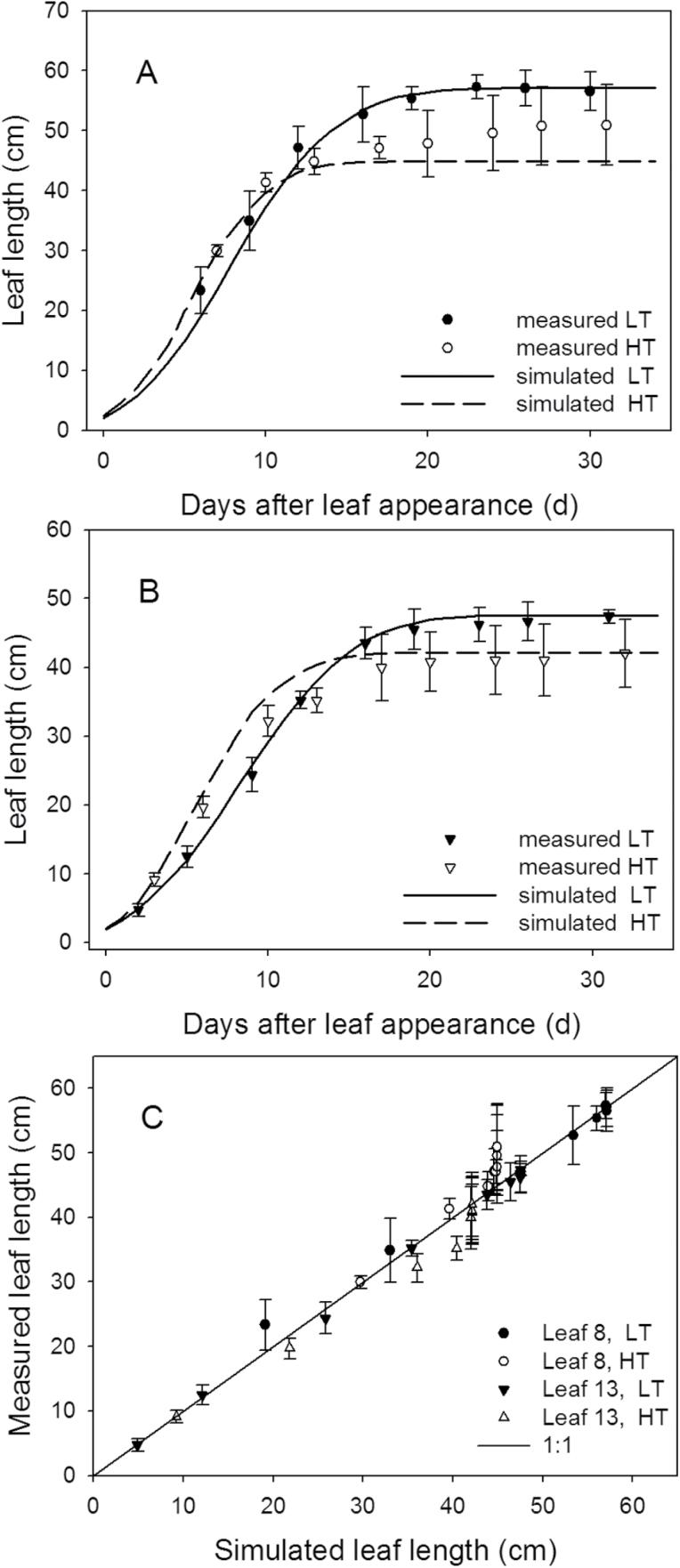
Measured (symbols) and simulated (lines) leaf lengths at rank 8 (A) and rank 13 (B) under 22/18 °C (LT, filled symbols) and 32/28 °C (HT, open symbols) day/night temperature conditions (experiment 5, *n*=4). Bars indicate standard errors. The solid line in (C) is the 1:1 line between simulated and measured data. By plotting all data, *y*=1.00*x*+0.04, *R* ^2^=0.97, *P*<0.001, the intercept was not different from zero and the slope was not different from one.

**Fig. 4.**
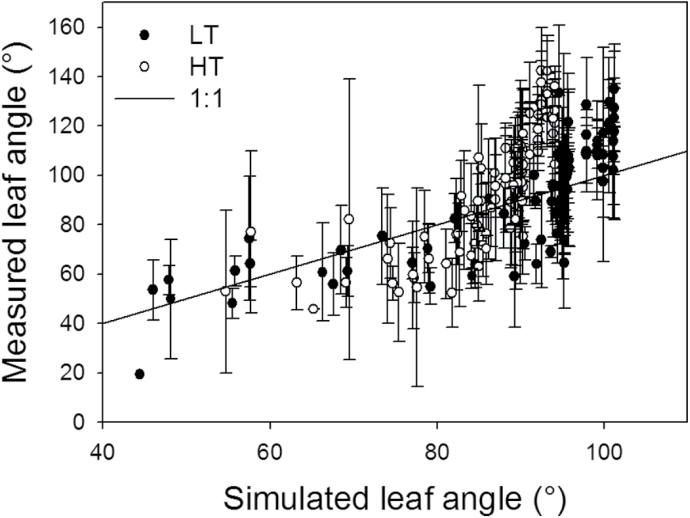
Measured and simulated leaf angles at 22/18 °C (LT, filled circles) and 32/28 °C (HT, open circles) day/night temperature conditions (experiment 5, *n*=4). Data were taken from the plants on 50, 56, 63, and 64 DAFLA Bars are standard errors. The solid line is the 1:1 line between simulations and measurements.

The simulation showed that maximum leaf elongation rates, *E*_l,max_ (cm d^–1^), of the leaves at rank 8 and 13 were lower in the 22/18 °C day/night temperature treatment (LT) than in the 32/28 °C day/night temperature treatment (HT; Fig. 2) and the time (days) taken to complete leaf growth was longer in the LT than in the HT treatment (Fig. 3A, B). Leaves of plants grown under LT were larger than of those grown under HT conditions, for both rank 8 and 13. Furthermore, the random factor in the model only resulted in a very slight difference (<0.1cm) between simulations. Therefore, in Fig. 3A and B, the results of a single simulation are presented which are in good agreement with measured leaf lengths (Fig. 3C). The accuracies for both ranks and temperature conditions were >90%, but the model predicted leaf growth under LT better than under HT for both ranks (8 and 13), as indicated by the RMSDs and bias (Table 1). Simulated internode growth was faster under high than low temperatures in the early phase (Supplementary Fig. S7). However, final internode length differed only slightly between these two conditions. Simulated leaf angles under LT and HT were well in accordance with measurements (Fig. 4), with 76% and 80% accuracies, respectively (Table 1).

**Table 1.** Statistical analysis for the comparison between simulated and measured data for organ and canopy levels over the whole duration of leaf and plant growth at 22/18 °C (LT) and 32/28 °C (HT) day/night temperatures

Traits	Figure	LT			HT		
		RMSD	Bias	Accuracy (%)	RMSD	Bias	Accuracy (%)
*L*_l_ at rank 8	Fig. 3A	2.04	0.98	96	3.25	2.66	93
*L*_l_ at rank 13	Fig. 3B	0.82	–0.59	97	2.51	–2.03	92
*L*_i_ at rank 8	Supplementary Fig. S7	0.38	–0.07	89	0.35	–0.08	91
Leaf angle	Fig. 4	21.35	16.46	76	17.05	7.54	80
Leaf number	Fig. 5A	0.96	0.89	96	0.86	–0.26	97
Plant height	Fig. 5B	5.36	–0.18	95	3.39	–2.65	97
Total leaf area	Fig. 5C	1038	649	95	765	–133	95
Shoot dry mass	Fig. 5D	44.94	37.84	85	17.39	–1.55	93

*L*_l_, leaf length; *L*_i_, internode length; RMSD, root mean square deviation in equation 14a.

### Model evaluation at the canopy level

**Fig. 5.**
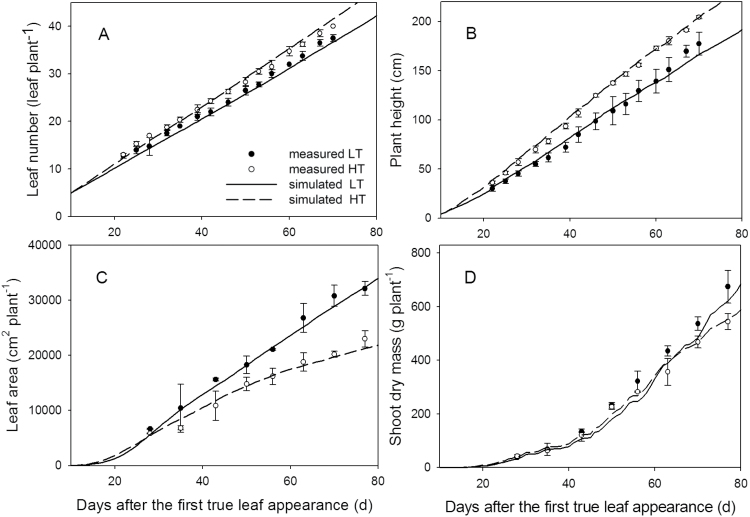
Comparison between simulated and measured leaf number (A), plant height (B), total leaf area (C), and shoot dry mass (D) at 22/18 °C (LT, filled circles) and 32/28 °C (HT, open circles) day/night temperature conditions (experiment 5, *n*=4). Bars are standard errors. Lines represent the averages of simulated data under LT (solid line) and HT conditions.

Plants exposed to HT produced more leaves than those grown under LT, but the differences between temperature regimes were less than three leaves (Fig. 5A). For both conditions, the model predicted leaf number with accuracies >95% (Table 1). Plant height at LT was 74–80% of that at HT (Fig. 5B). The simulated plant heights were in good agreement with the measured data, with differences not exceeding 10%. Both measured and simulated results showed that plants under LT had larger total leaf area than those under HT throughout. At the last measurement (77 DAFLA), HT plants had total leaf areas amounting to only 65% and 66% of the leaf area of the plants at LT for simulation and measurement, respectively (Fig. 5C). The accuracies of the model at both temperatures were 95%. Total shoot dry mass, *W*_s_, at LT was 15–20% higher than at HT from day 50 on (Fig. 5D). The model predicted *W*_s_ at HT with a 93% accuracy but less exactly at LT (Fig. 5D). After 77 DAFLA, the model overestimated *W*_s_ at HT by 2% and underestimated it at LT by 8%. The standard errors of the simulated total leaf area and *W*_s_ between simulations were very small (<1%). Therefore, only average values are shown in Fig. 5C and D. Interestingly, simulations of shoot dry mass with (Fig. 5D) and without Supplementary Fig. S8 at *JXB* online) the temperature effect on light use efficiency [the term {1–κ[*T*(*t*)–T*]^2^} in equation 12) were not greatly different. Therefore, temperature effect on light use efficiency was excluded for the analyses of morphological traits.

### Analyses of morphological traits

**Fig. 6.**
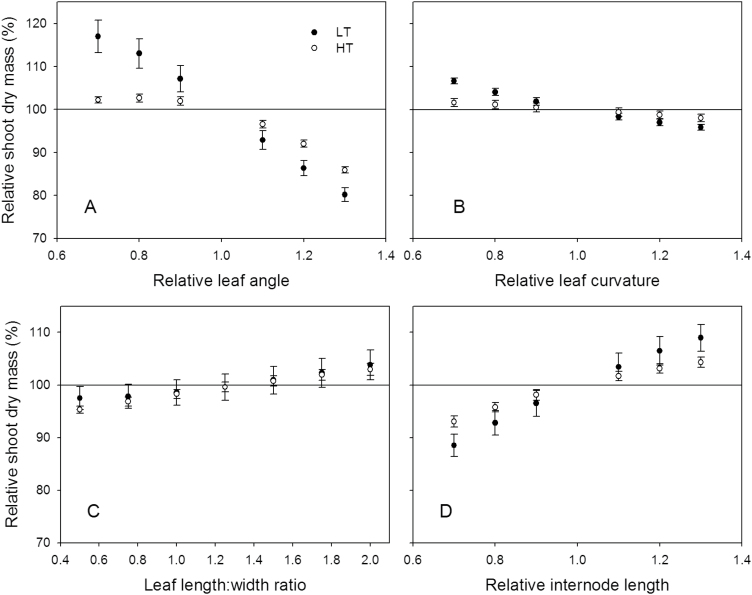
The predicted influence of the leaf angle (A), leaf curvature (B), leaf length and width ratio (C), and internode length (D) on shoot dry mass on 77 DAFLA at 22/18 °C (LT, filled circles) and 32/28 °C (HT, open circles) day/night temperature conditions. The reference values for relative leaf angle, leaf curvature, and internode length were 1. The reference value for leaf length and width ratio was 1.33. Simulated shoot dry mass on 77 DAFLA with the reference values was set to 100%.

At LT, decreasing leaf angles by 30% resulted in a 17% increase in *W*_s_ (Fig. 6A) on 77 DAFLA. Interestingly, the corresponding increase at HT was only 2.2%. In contrast, increases in leaf angle reduced dry mass production: a 30% increase in leaf angle resulted in 19.8% and 14.1% reduction of *W*_s_ at LT and HT, respectively. In comparison with the leaf angle, leaf curvature and leaf length:leaf width ratio had less effect on *W*_s_. For example, a decrease in leaf curvature by 30% increased shoot dry mass by 6.6% and 1.5% at LT and HT, respectively (Fig. 6B); and plants with narrow leaves (e.g. leaf length:width ratio=2) had an up to 3.8% higher *W*_s_ (Fig. 6C). Shorter internodes had negative effects on *W*_s_; thus *W*_s_ of the plants with 30% shorter internodes was reduced by 11.5% and 6.9% at LT and HT, respectively (Fig. 6D).

**Fig. 7.**
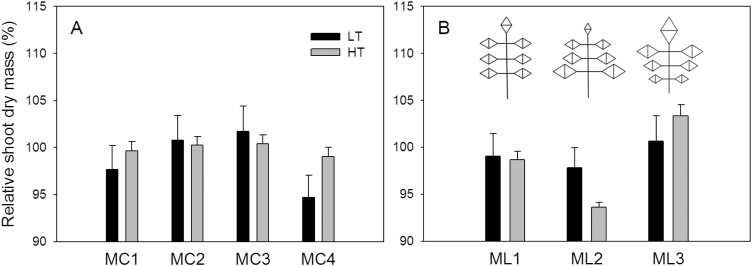
The predicted influence of the leaf curvature angle ratio (A) and leaflet arrangement (B) on total shoot dry mass on 77 DAFLA at 22/18 °C (LT, black bar) and 32/28 °C (HT, grey bar) day/night temperature conditions. The reference ratio of curvature angles, α_1_:α_2_:α_3_ (Fig. 1), was 1:2:2; MC1=1:1:1; MC2=1:1:2; MC3=1:2:3; and MC4=2:1:1. Reference area ratio of leaflet 1:leaflet 2:leaflet 3:terminal leaflet was 0.12:0.17:0.13:0.16; ML1=0.143:0.143:0.143:0.142; ML2=0.2:0.15:0.11:0.08; and ML3=0.08:0.15:0.17:0.2.

**Fig. 8.**
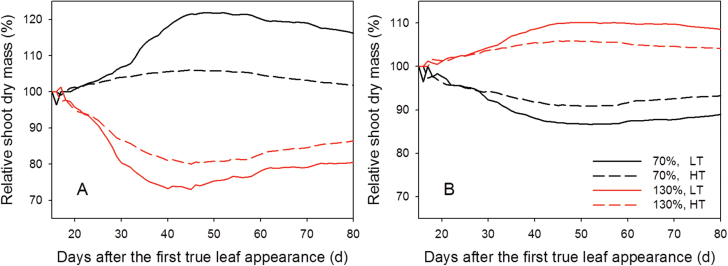
The simulated influence of the leaf angle (A) and internode length (B) on shoot dry mass at 22/18 °C (LT, solid lines) and 32/28 °C (HT, dashed lines) day/night temperature conditions. Black and grey lines represent that the morphological traits are 70% and 130% of the reference values, respectively. (This figure is available in colour at *JXB* online.)

Changes in ratios of the leaf curvature angles affected *W*_s_ to a lesser extent, at both LT and HT (Fig. 7A). The strongest reduction of 6% occurred in plants with leaves more curved at the leaf base (MC4, α_1_:α_2_:α_3_=2:1:1). For leaflet arrangement, the ML1 scenario, where all leaflets were equal in size, had nearly the same shoot dry mass as the reference leaflet arrangement (Fig. 7B). In the ML2 scenario, where leaflet 1 was larger and the terminal leaf was smaller, *W*_s_ was reduced by 2.2% and 6.4% under LT and HT conditions, respectively. In the ML3 scenario, where leaflet 1 was smaller and the terminal leaf was larger, *W*_s_ at HT increased slightly by 3.4%. Among all the morphological traits tested by the analyses in this study, leaf angle and internode length were the traits having the strongest effects on *W*_s_ (Fig. 8A, B). These effects were most prominent between 25 and 40 DAFLA when the LAI was between 0.4 and 1 (Fig. 5C). All the results from the analyses suggested that, in general, changes in morphological traits at HT had less influence on *W*_s_ than at LT.

### Canopy light interception

Simulated light transmission (*Q*_t_/*Q*_0_) through the canopy decreased with time regardless of temperature conditions. HT allowed more light to be transmitted through the canopy than LT (Table 2). Except on 28 DAFLA, a 30% decrease in leaf angle, θ, reduced *Q*_t_/*Q*_0_ by ~10% at LT but had no effect at HT (Table 2). Conversely, an increase in leaf angle by 30% increased the *Q*_t_/*Q*_0_ by 15–20% at both LT and HT. The light extinction coefficient, *k*, decreased over time and was higher at LT than at HT. At LT, a decrease in leaf angle increased *k* and an increase in θ reduced *k*. At HT, a decrease of θ had no effect on *k* and an increase in θ reduced *k*. Interestingly, changes in internode length had no effects on *Q*_t_/*Q*_0_ and *k*. Further data about the effects of changing architectural traits on canopy light interceptions and *k* can be found in Supplementary Table S4 at *JXB* online.

**Table 2.** Influence of leaf angle and internode length on light transmission through the simulated tomato canopy (Q_t_/Q_0_), light extinction coefficient (k), and on different days expressed in days after appearance of the first true leaf (DAFLA) at 22/18 °C (LT) and 32/28 °C (HT) day/night temperature conditionsNumbers are means with standard error in parentheses.

Scenario	DAFLA	LT		HT	
		*Q*_t_/*Q*_0_	*k*	*Q*_t_/*Q*_0_	*k*
Reference					
	28	0.67 (0.01)	0.71 (0.03)	0.71 (0.02)	0.63 (0.05)
	43	0.44 (0.02)	0.58 (0.04)	0.50 (0.02)	0.59 (0.04)
	56	0.35 (0.02)	0.49 (0.02)	0.41 (0.02)	0.54 (0.03)
	63	0.32 (0.02)	0.45 (0.02)	0.39 (0.02)	0.52 (0.02)
70% leaf angle					
	28	0.70 (0.01)	0.62 (0.01)	0.75 (0.01)	0.52 (0.03)
	43	0.35 (0.02)	0.73 (0.04)	0.51 (0.42)	0.56 (0.03)
	56	0.25 (0.02)	0.64 (0.03)	0.42 (0.01)	0.53 (0.02)
	63	0.22 (0.01)	0.60 (0.02)	0.40 (0.01)	0.50 (0.02)
130% leaf angle					
	28	0.83 (0.01)	0.33 (0.02)	0.81 (0.01)	0.39 (0.01)
	43	0.62 (0.01)	0.33 (0.01)	0.66 (0.01)	0.35 (0.01)
	56	0.53 (0.02)	0.29 (0.01)	0.58 (0.01)	0.33 (0.01)
	63	0.51 (0.01)	0.27 (0.01)	0.55 (0.01)	0.33 (0.01)
70% internode					
	28	0.68 (0.01)	0.68 (0.04)	0.68 (0.02)	0.70 (0.06)
	43	0.43 (0.03)	0.59 (0.05)	0.48 (0.02)	0.63 (0.04)
	56	0.35 (0.02)	0.49 (0.03)	0.40 (0.02)	0.56 (0.03)
	63	0.33 (0.02)	0.45 (0.03)	0.38 (0.02)	0.53 (0.03)
130% internode					
	28	0.68 (0.02)	0.68 (0.04)	0.73 (0.02)	0.58 (0.05)
	43	0.44 (0.02)	0.57 (0.03)	0.51 (0.02)	0.56 (0.03)
	56	0.35 (0.02)	0.49 (0.03)	0.42 (0.02)	0.53 (0.03)
	63	0.32 (0.01)	0.45 (0.02)	0.41 (0.02)	0.50 (0.03)

## Discussion

FSPMs are particularly suitable for studying structure-related research questions (Vos *et al.*, 2010; DeJong *et al.*, 2011; Poorter *et al.*, 2013). In comparison with traditional crop models, it requires more parameters to construct a dynamic FSPM (Evers *et al.*, 2010), but a precise and detailed description of canopy structure is a condition for the accurate evaluation of the sensitivity of canopy light interception and dry mass production to architectural traits (Song *et al.*, 2013). To ensure that the results from the analyses are plausible, the careful evaluation of the model is a prerequisite (Evers *et al.*, 2010; Evans, 2013).

### Evaluation of model performance

The simulated results, at both organ and canopy levels, were well in accordance with the measurements in the experiment for model evaluation (Figs 2–4). The accuracies of the model in predicting architectural traits were >90% (Table 1), except for the internode length under LT conditions (89%). The number of leaves at HT was slightly overestimated after 60 DAFLA (Fig. 5A). This may have resulted from day temperatures >30 °C (Supplementary Fig. S1 at *JXB* online). Probably, above 30 °C, the leaf appearance rate (*R*_l_) decreases slightly instead of maintaining *R*_lmax_ as was assumed (equation 6). Nevertheless, simulation and measurement were still in good agreement (Fig. 5A). The less accurate prediction of internode length was due to an overestimation of the internode elongation rate (Fig. 3), which was dependent on temperature and light quantity (equation 9). However, it has been shown that both light quantity and light quality (e.g. red:far red ratio) may affect internode growth (Ballaré, 2009). By using a dynamic FSPM, Kahlen and Stützel (2011) have demonstrated that introducing the effect of light quality on internode length may improve its prediction in cucumber. It will be interesting to study whether their approach can be used for predicting tomato internode length more accurately. The predicted shoot dry mass over time was similar to the measurements, but less satisfactory (86% and 90% accuracies for LT and HT conditions, respectively; Table 1). Nevertheless, it can be concluded that the model already has good performance in predicting dynamic plant architecture and dry mass production under LT and HT conditions.

### Temperature effects on canopy structure and light interception

An increase in temperature increased the leaf elongation rate, *E*_l_, between base temperature and optimum temperature. Above optimum temperature, further increasing temperatures would decrease the leaf elongation rate. This response of *E*_l_ to temperature in the present study followed a similar pattern to that found in other plant species (Parent and Tardieu, 2012). Although the leaf elongation rates at HT were higher than those at LT (Fig. 2), final leaf lengths at HT were 87% and 86% of those at LT for the leaves at ranks 8 and 13, respectively (Fig. 3). This is due to an ~5 d shorter duration of leaf growth at HT (Fig. 2). Whole-plant leaf area consists of two components: leaf number and leaf area. Although tomato plants produce more leaves at HT than at LT, this was not sufficient to compensate for the smaller single leaf area. Consequently, plants at LT had more leaf area. Furthermore, plants at HT had longer internodes than at LT (Fig. 5B; Supplementary Fig. S7 at *JXB* online). The differences in leaf area and internode length between LT and HT resulted in the change in canopy structure. Smaller leaves and longer internodes at HT constructed a canopy with lower crown density (canopy surface area:canopy leaf area) and probably higher leaf dispersion (less clumped leaves; Duursma *et al.*, 2012). This resulted in a higher transmittance of light through the canopy but a larger light extinction coefficient, *k* (Table 2). A larger *k* value indicates that more light is intercepted per unit leaf area (Duursma *et al.*, 2012). This might explain why plants at HT produced more dry matter per unit leaf area than those at LT (Fig. 5C, D). Therefore, the higher shoot dry mass in LT was not in proportion to the larger plant leaf area.

There are two possible reasons for the differences of *k* between canopies under low and high temperature regimes. The first could be a different leaf angle distribution in the middle layer of the canopy. In the top of the canopy, leaf angle distribution between the two canopies was similar. However, the leaf angle in the middle of the canopy at HT was more horizontal (85–90 °) than at LT (~100 °; Supplementary Fig. S9A at *JXB* online). From the model analyses, a smaller leaf angle increases the light absorption from the canopy, increasing *k* (Table 2) and shoot dry mass (Fig. 6A). The second reason might be leaf curvature. Similarly, the curvatures of leaves in the upper layer between the two temperature conditions were not different. Again, the leaves in the middle section of the plants in the LT treatment were ~20 ° more curved than those in the HT treatment (Supplementary Fig. S9B). More curvature would increase mutual leaf shading due to overlapping; this leads to a reduction in area available for light interception in the canopy at LT. Therefore, a decrease in leaf curvature would be associated with an increase in shoot dry mass (Fig. 6B).

### Potential impacts of architectural traits on dry mass production

The present results strongly suggest that (i) there are substantial impacts of plant architectural traits on dry mass production and canopy light interception, and leaf angle and internode length have the strongest impacts; (ii) there are interactions between these effects and temperature; and (iii) for dry mass production, canopies with a more clumped structure are more sensitive to changes in architectural traits.

Clearly, all architectural traits, leaf angle (Fig. 6A), leaf curvature (Fig. 6B), leaf length:width ratio (Fig. 6C), internode length (Fig. 6D), curvature ratio (Fig. 7A), and leaflet arrangement (Fig. 7B), affect light interception and dry mass production of tomato. According to the present results, leaf angle and internode length would affect plant productivity more than other morphological changes, which is in accordance with the results of Sarlikioti *et al.* (2011) and Song *et al.* (2013). For leaf angle, it has been suggested that an ideal plant for light interception has small and vertical leaves in the upper part, which allow more light to penetrate to the lower part where leaves are large and horizontal (Zhu *et al.*, 2010). This could explain the increase in shoot dry mass as leaf angle decreased (Fig. 6A). However, the magnitudes of the results were quite discrepant from the values reported in the literature. Sarlikioti *et al.* (2011) and Song *et al.* (2013) reported that changes in leaf angle and internode length could increase or decrease canopy photosynthesis by 3–7%, but the present results suggested that these changes could influence the shoot dry mass by up to 20% (Fig. 6A). A simple explanation is that the canopy models used in these two studies were static models and the simulations were only run for 1 d and for several specific environmental conditions. In reality, the increase in canopy photosynthesis due to changes in architectural traits on one day affects canopy growth and therefore light interception of the next day, so that this self-enforcing effect has to be taken into account, which can be done only in dynamic models such as that presented here. This effect could be observed between 20 and 40 DAFLA (Fig. 8). Moreover, in reality, plants grow in a fluctuating environment and canopy structure changes daily. Since there are strong interactions between canopy structure and light interception, and the relationship, at both leaf and organ levels, between light interception and photosynthesis rate is not linear (Warren-Wilson *et al.*, 1992; Sarlikioti *et al.*, 2011; Zhu *et al.*, 2012; Song *et al.*, 2013; von Caemmerer, 2013), the results of running simulations for one specific condition may not be valid to generalize the architectural effects to canopy photosynthesis.

Longer internodes could increase dry mass production (Figs 6D, 8B) because increased internode length would increase the distance between leaves and hence reduce canopy density and self-shading, which improve light interception (Takenaka, 1994; Sarlikioti *et al.*, 2011). However, no difference in light transmittance and light extinction coefficient was found between the reference and ±30% internode length (Table 2). This suggests that a canopy with plants having longer internodes does not intercept more light, but the light might be better distributed in the canopy.

Analyses of architectural traits showed that dry mass production at HT, in most cases, was less influenced by changes in architectural traits than at LT (Figs 6–8). Dry mass production at LT and HT was modelled by the same parameter set and light intensity above the canopy. As discussed above, canopy structure at HT was less clumped, had lower crown density, and the leaves in the canopy were less self-shaded. Therefore, the degree of improvement in light distribution within the canopy structure at HT through a better leaf distribution would be less than in a more closed canopy structure. This idea can be supported by the finding that light interception is more sensitive to canopy structure when the crown density is high and the leaves in the canopy are more clumped (Duursma *et al.*, 2012). These results imply that there are interactions between temperature regime and the impacts of architectural traits on dry mass production. Another interesting question is whether the high temperature always results in a more open canopy structure for different species; however, answering this question is beyond the scope of this study.

It is important to emphasize that not only the precise description of the canopy structure, but also the dynamic changes in canopy architecture and environment over time must be taken into account when quantifying the potential impacts of the architectural traits on light interception and, consequently, on plant productivity. It is concluded that dynamic FSPMs may serve as a suitable tool to achieve this objective. Further studies using dynamic FSPMs may help in designing the ‘ideotype’ and ideal canopy structure for different environmental conditions.

## Supplementary data

Supplementary data are available at *JXB* online.

Figure S1. Fluctuation of daily global radiation, day temperature, and VPD in experiment 5.

Figure S2. Leaf number *N*_l_ over time at different temperatures *T*, and leaf appearance rate (LAR) in relation to temperature

Figure S3. Effect of vapour pressure deficit and temperature on maximum leaf elongation rate.

Figure S4. Time courses of normalized leaf elongation rate at different temperature regimes of the leaves at rank 8 (experiment 1).

Figure S5. Normalized function of leaf rank effect on final leaf length.

Figure S6. Leaf angle and leaf curvature with leaf length *L*_l_.

Figure S7. Measured and simulated internode lengths at rank 8 at 22/18 °C and 32/28 °C day/night temperature conditions.

Figure S8. Comparison between simulated (without temperature effect on light use efficiency) and measured shoot dry mass at 22/18 °C and 32/28 °C day/night temperature conditions.

Figure S9. Leaf angle and leaf curvature along the leaf rank on day 77 after appearance of the first true leaf at 22/18 °C and 32/28 °C day/night temperature conditions.

Table S1. Summary of experimental conditions.

Table S2. Schedule for experiment cultivation.

Table S3. Values of all parameters used in the model and their comparable values reported in the literature.

Table S4. Influence of leaf curvature and leaf length:width ratio on light transmission through the simulated tomato canopy, light extinction coefficient, and on different days expressed in days after appearance of the first true leaf at 22/18 °C and 32/28 °C day/night temperature conditions.

Supplementary Data
