# Supplementary material for: Quantification of the effects of architectural traits on dry mass production and light interception of tomato canopy under different temperature regimes using a dynamic functional–structural plant model
Source: J Exp Bot. 2014 Sep 2;65(22):6399–410. doi: 10.1093/jxb/eru356 (PMC4246178; doi:10.1093/jxb/eru356)
Supplement: Supplementary Data [file supp_65_22_6399__index.html]

Quantification of the effects of architectural traits on dry mass production and light interception of tomato canopy under different temperature regimes using a dynamic functional–structural plant model — Quantification of the effects of architectural traits on dry mass production and light interception of tomato canopy under different temperature regimes using a dynamic functional–structural plant model — Supplementary Data 

# Quantification of the effects of architectural traits on dry mass production and light interception of tomato canopy under different temperature regimes using a dynamic functional–structural plant model

## Supplementary Data

Data files

**Files in this Data Supplement:**

- Supplementary Data - Supplementary Data
