# Supplementary material for: Quantification of the effects of architectural traits on dry mass production and light interception of tomato canopy under different temperature regimes using a dynamic functional–structural plant model
Source: J Exp Bot. 2014 Sep 2;65(22):6399–410. doi: 10.1093/jxb/eru356 (PMC4246178; doi:10.1093/jxb/eru356)
Supplement: Supplementary Data [file supp_eru356_Tomato_FSPM_morphology_manuscrip_SI_rev.pdf]

TABLE S1 Summary of experimental conditions.

Expt.	Location	Set conditions			
		Day/night temperature (°C)	VPD (kPa)	PAR (µmol m^{-2} s^{-1})	CO_2 (ppm)
1	Growth chamber	17/13, 22/18, 26/22 & 30/26	0.8	300	380
2	Growth chamber	22/18	0.4, 0.8 & 1.2	300	380
3	Growth chamber	22/18	0.8	300, 500 & 700	380
4	Greenhouse	22/18	-	-	ambient
5	Greenhouse	22/18	-	-	ambient
		& 32/28	-	-	ambient

TABLE S2. Schedule for experiment cultivation

Experiment	Sowing	Transplanting to large rock wool cubes	Transplanting to growth chambers or greenhouses
1	5 Aug 2008	18Aug 2008	22 Aug 2008
2	23 Oct 2008	5Oct 2008	8 Oct 2008
3	23 Feb 2009	3 Mar 2009	9 Mar 2009
4	11 May 2009	20 May 2009	30 May 2009
5	22 Mar 2010	1 Apr 2010	14 Apr 2010

TABLE S3. Values of all parameters used in the model and their comparable values reported in the literature. Numbers in brackets denote equation numbers.

Parameter (Eqn.)	Our value	Unit	Value in the literature
T_{bl} (2a & b)	6.8	°C	5.9°C in tomato in Heuvelink (1995)<br>6-10°C in tomato in Calado and Portas (1987)<br>8°C in tomato in Najla *et al.* (2009)
$a_{El,max}$ (2a & b)<br>$b_{El,max}$ (2a & b)<br>T_{opt} (2a & b)<br>$TS_{l,max}$ (3)<br>h_l (3)	0.318<br>-0.0291<br>28<br>135<br>82	-<br>-<br>°C<br>°Cd<br>-	For $a_{El,max}$, $b_{El,max}$, $TS_{l,max}$ and h_l, no comparable parameters were found for tomato in the literature. However, Reymond *et al.* (2003) reported in maize that $a_{El,max}$ and $b_{El,max}$ range between 0.3 — 0.6 and -0.14 — -0.06, respectively. We do not find T_{opt} for tomato, but this parameter is between 25-30°C for most of plant species (Parent and Tardieu, 2012)
R_{max} (4)<br>h_r (4)	9<br>6	-<br>-	R_{max} and h_r described the effect of leaf rank on leaf lengh. Here we compare the effect of leaf rank on final leaf length in different species. Points in the graph are derived from published data.<br>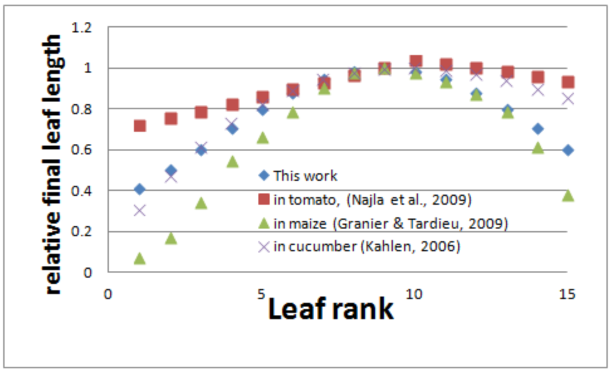
a_{Al} (5)<br>g (5)	0.92<br>2.4	-<br>-	Schwarz and Kläring (2001) reported several sets of a_{Al} and g in tomato. a_{Al} was between 0.26-0.42 and g was between 2.03-2.70.
a_r (6a)<br>b_r (6a)	0.37<br>0.63	-<br>-	For a_r and b_r, no comparable parameters were found for tomato in the literature.
a_θ (7)<br>b_θ (7)<br>a_{Cl} (8a)<br>b_{Cl} (8a)<br>a_{1Cl} (8b)<br>b_{1Cl} (8b)	114.6<br>0.04<br>200<br>2.6<br>-161<br>4.75	-<br>-<br>°<br>° cm^{-1}<br>°<br>° cm^{-1}	Parameters in Eqn 7 and 8 describe the relationship between leaf length and leaf angle (θ). Najla *et al.* (2009) assumed that θ equal to 60° for all leaves, which is not realistic; Sarlikioti *et al.* (2011) reported that θ ranges from 75°-125°; and de Visser *et al.* (2014) assumed that θ ranges from 60°-90°. The simulated leaf angles in this work range between 45°-100° (Fig. 4), which are in accordance with the data in the literature.

T_{bi} (9)	10	°C	6-10°C in tomato in Calado and Portas (1987)<br>8°C in tomato in Najlia *et al.* (2009)
$a_{Ei,max}$ (9)<br>$b_{Ei,max}$ (9)<br>$TS_{i,max}$ (10)<br>h_i (10)	0.063<br>0.0000526<br>85<br>35	-<br>-<br>°Cd<br>-	For $a_{Ei,max}$, $b_{Ei,max}$, $TS_{i,max}$ and h_i, no comparable parameters were found for tomato in the literature. Najla *et al.* (2009) simply described the growth of internode length increase 1.1 mm per °Cd.
a_{Di} (11)<br>b_{Di} (11)	1.33<br>0.0011	cm<br>cm °Cd^{-1}	For a_{Di} and b_{Di}, no comparable parameters were found for tomato in the literature.
μ (13)	0.87		0.89 in tomato in Maggio *et al.*, 2007<br>0.70-0.91 in tomato in Ågren and Franklin (2003)
Phy	144±10	°	130° in tomato in de Visser *et al.* (2014)<br>137.5° in tomato in Tomato Anatomy<br>144° in tomato in Najlia *et al.* (2009)
$TS_{l,sen}$	980	°Cd	8-10 weeks in tomato in John *et al.* (1995). We recalculated $TS_{l,sen}$ according to the experimental condition in John *et al.* (1995) (22/18°C day night temperature). $TS_{l,sen}$ ranges between 851-1064 °Cd in this experiment.

Ågren GI and Franklin O. 2003. Root: shoot ratios, optimazation and nitrogen productivity. *Annals of Botany* **92**, 795-800.

Calado AM and Portas CM. 1987. Base-temperature and date of planting in processing tomatoes. *Acta Horticalturae* **200**, 185-188.

de Visser PHB, Buck-Sorlin GH and van der Heijden GWAM. 2014. Optimizing illumination in the greenhouse using a 3D model of tomato and a ray tracer. *Frontier in Plant Science* **5**, 48. doi:10.3389/fpls.2014.00048

Granier C and Tardieu. 2009. Multi-scale phenotyping of leaf expansion in response to environmental changes: the whole is more than the sum of parts. *Plant, Cell and Environment* **32**, 1175-1184.

Heuvelink E. 1995. Growth, development and yield of a tomato crop: periodic destructive measurements in a greenhouse. *Scientia Horticulturae* **61**, 77-99.

John, I., Drake, R., Farrell, A., Cooper, W., Lee, P., Horton, P. and Grierson, D. 1995. Delayed leaf senescence in ethylene-deficient ACC-oxidase antisense tomato plants: molecular and physiological analysis. *The Plant Journal*, **7**, 483–490. doi: 10.1046/j.1365-313X.1995.7030483.x

Kahlen K. 2006. 3D Architectural modelling of greenhouse cucumber (*Cucumis sativus* L.) using L-systems. *Acta Horticalturae* **718**, 51-59.

Maggio A, Raimondi G, Martino A, Pascale S de. 2007. Salt stress response in tomato beyond the salinity tolerance threshold. *Environmental and Experimental Botany* **59,** 276–282.

Najla S, Vercambre G, Pages L, Grasselly D, Gautier H, Genard M. 2009. Tomato plant architecture as affected by salinity: Descriptive analysis and integration in a 3-D simulation model. *Botany* **87,** 893–904.

Parent B, Tardieu F. 2012. Temperature responses of developmental processes have not been affected by breeding in different ecological areas for 17 crop species. *New Phytologist* **194,** 760–774.

Reymond M, Muller B, Leonardi A, Charcosset A, Tardieu F. 2003. Combining quantitative trait loci analysis and an ecophysiological model to analyze the genetic variability of the responses of maize leaf growth to temperature and water deficit. *Plant Physiology* **131,** 664–675.
Sarlikioti V, Visser PHB de, Buck-Sorlin GH, Marcelis LFM. 2011. Exploring the spatial distribution of light interception and photosynthesis of canopies by means of a functional–structural plant model. *Annals of Botany* **107,** 875-883.
Schwarz D and Kläring PH. 2001. Allometry to estimate leaf area of tomato. *Journal of Plant Nutrition* **24**, 1291-1309, DOI: 10.1081/PLN-100106982

Table. S4 Influence of leaf curvature and leaf length:width ratio on light transmission through the simulated tomato canopy (Q_t/Q_0), light extinction coefficient (k), and on different days expressed in days after appearance of the first true leaf (DAFLA) at 22/18°C (LT, black bar) and 32/28°C (HT, grey bar) day/night temperature conditions. Numbers are means with standard error in parentheses.

		LT		HT	
Scenario	DAFLA	Q_t/Q_0	k	Q_t/Q_0	k
70% leaf curvature					
	28	0.66 (0.01)	0.75 (0.03)	0.70 (0.02)	0.64 (0.05)
	43	0.36 (0.02)	0.70 (0.04)	0.48 (0.02)	0.62 (0.04)
	56	0.28 (0.02)	0.59 (0.03)	0.40 (0.02)	0.56 (0.03)
	63	0.25 (0.02)	0.55 (0.03)	0.38 (0.01)	0.54 (0.02)
130% leaf curvature					
	28	0.70 (0.01)	0.64 (0.04)	0.71 (0.02)	0.62 (0.05)
	43	0.47 (0.02)	0.53 (0.03)	0.52 (0.02)	0.56 (0.04)
	56	0.39 (0.02)	0.44 (0.03)	0.44 (0.02)	0.50 (0.03)
	63	0.36 (0.01)	0.40 (0.02)	0.42 (0.02)	0.48 (0.02)
length:width = 0.5					
	28	0.71 (0.02)	0.60 (0.04)	0.75 (0.02)	0.52 (0.05)
	43	0.47 (0.02)	0.53 (0.03)	0.55 (0.02)	0.41 (0.02)
	56	0.38 (0.02)	0.45 (0.03)	0.46 (0.02)	0.36 (0.02)
	63	0.35 (0.02)	0.42 (0.02)	0.44 (0.02)	0.33 (0.01)
length:width = 2.0					
	28	0.65 (0.02)	0.75 (0.04)	0.68 (0.02)	0.70 (0.06)
	43	0.40 (0.03)	0.63 (0.05)	0.48 (0.03)	0.64 (0.05)
	56	0.31 (0.01)	0.55 (0.02)	0.38 (0.01)	0.59 (0.02)
	63	0.28 (0.02)	0.50 (0.02)	0.37 (0.02)	0.55 (0.03)

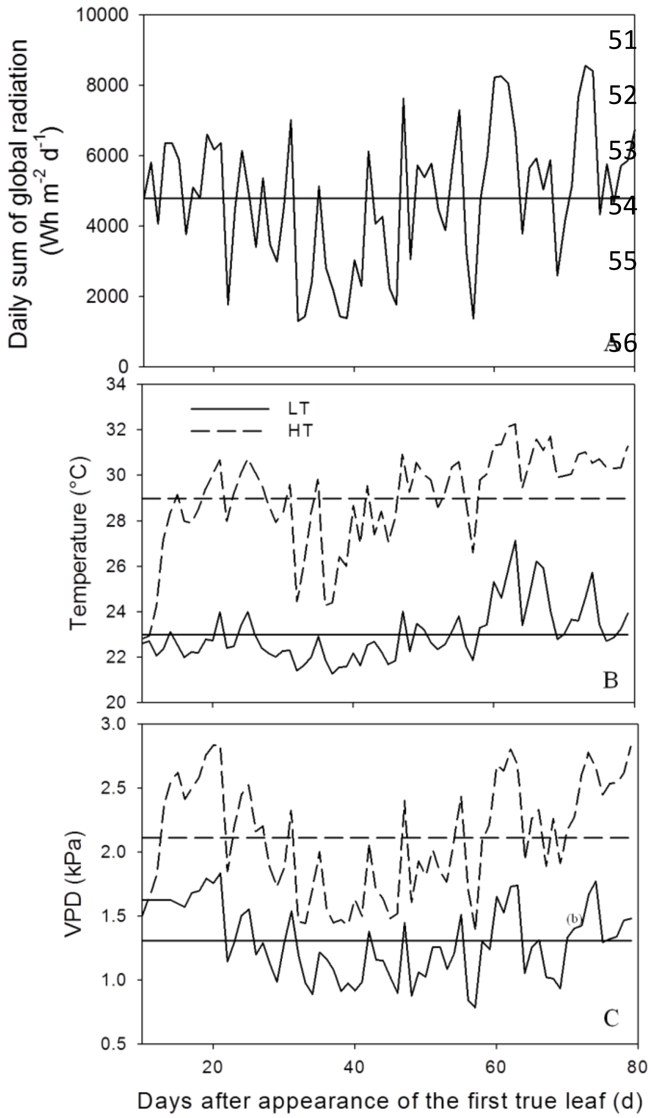

Fig. S1. Fluctuation of daily global radiation (A), day temperature (B) and VPD (C) in Expt. 5. Horizontal limes represent the mean values. Solid and dashed lines represent data at 22/18°C (LT) and 32/28°C (HT) day/night temperature conditions.

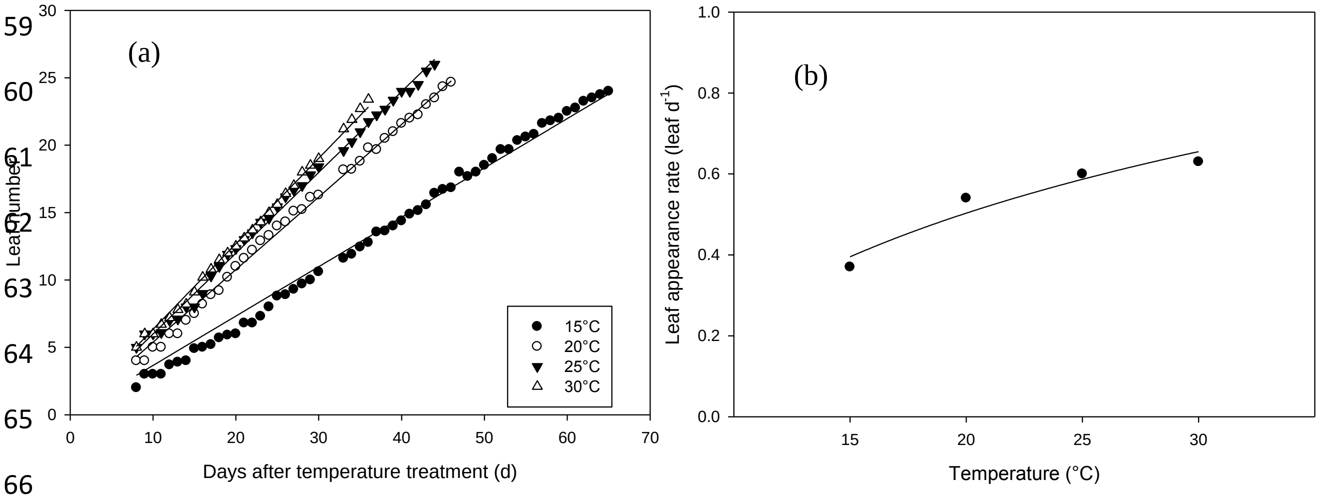

FIG. S2. (a) Leaf number N_l over time at different temperatures T (a). Symbols represent measured data (Expt. 1). Each point represents the mean value of six measurements. Lines resulted from linear regression analysis. At 15°C, $N_l = 0.37 \cdot T$ with $R^2 = 0.99$; at 20°C, $N_l = 0.54 \cdot T$ with $R^2 = 0.99$; at 25°C, $N_l = 0.6 \cdot T$ with $R^2 = 0.99$ and at 30°C, $N_l = 0.63 \cdot T$ with $R^2 = 0.99$. (b) Leaf appearance rate (LAR) in relation to temperature. Symbols represent the slopes from Fig 3a. LAR = $0.37 \cdot \ln(T) - 0.63$, $R^2 = 0.96$.

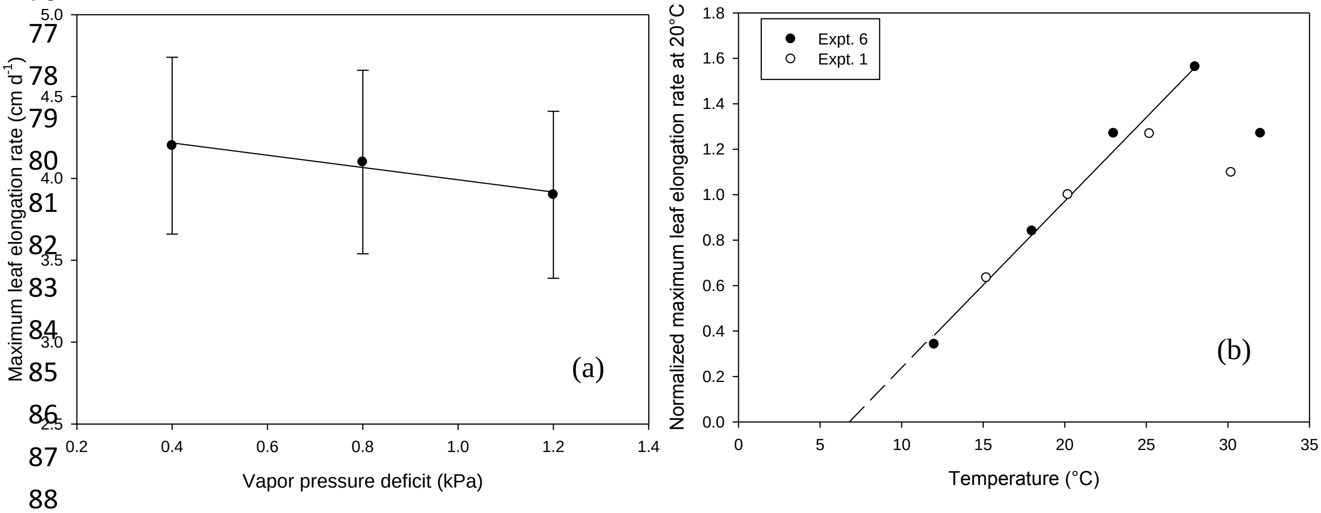

FIG . S3. (a) Effect of vapor pressure deficit, VPD, on maximum leaf elongation rate, $E_{l,max}$, of the leaves at rank 8. Each point is the mean value of five measurements (Expt. 2). $E_{l,max}$ = (4.73 – 0.37·VPD) with R^2 = 0.96. Bars represent standard deviations. (b) Effect of temperature on maximum leaf elongation rate, $E_{l,max}$. Data of two experiments were normalized at 20°C. Open circles represent data of Expt. 1. Closed circles represent data of Expt. 6. The solid line is linear regression over a range of temperatures from 12°C to 28°C with R^2=0.98. The dashed line is an extrapolated line from the linear regression resulting in the base temperature for leaf growth of 6.8°C.

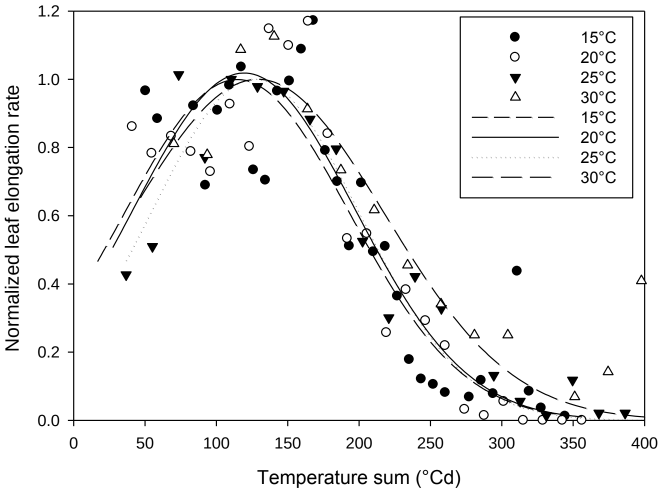

FIG. S4. Time courses of normalized leaf elongation rate at different temperature regimes of the leaves at rank 8 (Expt. 1). Symbols represent measured data and lines are fitted curves.

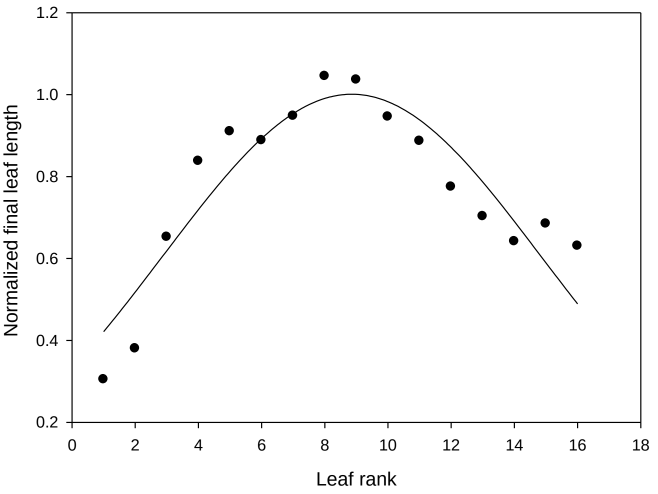

FIG. S5. Normalized function of leaf rank effect on final leaf length. Data were derived from Expt. 1 at 20°C treatment (n=3). Line is fitted curve with the bell shaped function, $y = \exp(-0.5 \cdot ((rank-9)/5.97)^2)$ and $R^2=0.84$.

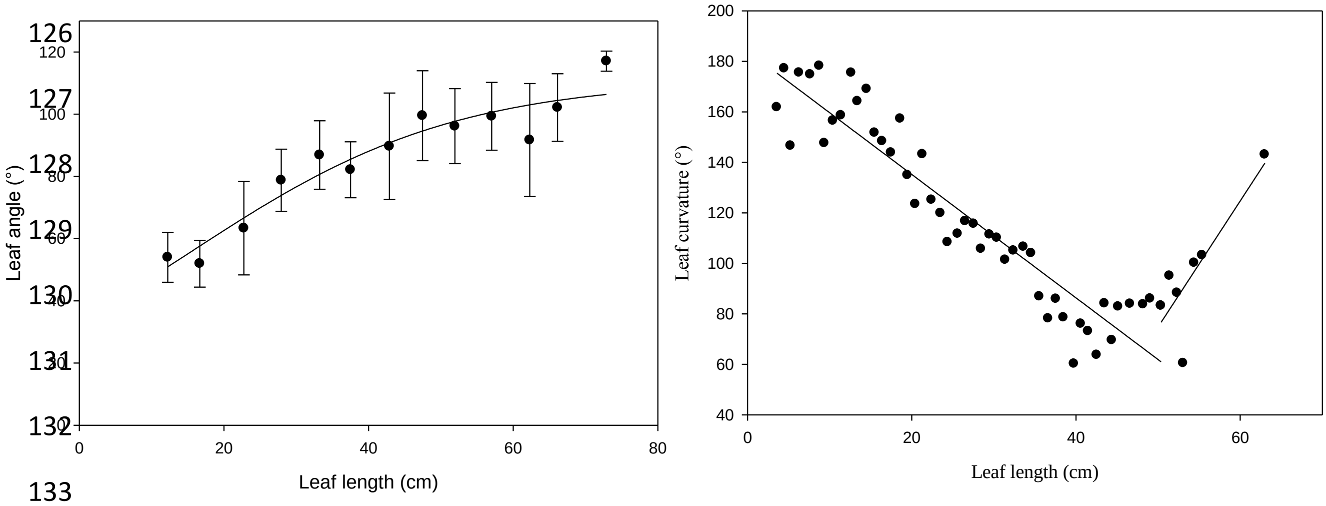

FIG. S6. Leaf angle (a) and leaf curvature (b) with leaf length L_l. Data were derived from digitizing data of Expt. 4 at different leaf ranks. The points are measured data (n=10). The lines are fitted curves with leaf angle, $ANG = 114.6 \cdot (1-\exp(-0.04 \cdot L_l))$, $R^2 = 0.95$ and leaf curvature, $CUR = 200-2.6 \cdot L_l$, $R^2=0.67$ for $L_l \leq 50$ and $CUR = -161 + 4.75.L_l$, $R^2= 0.95$ for $L_l>50$.

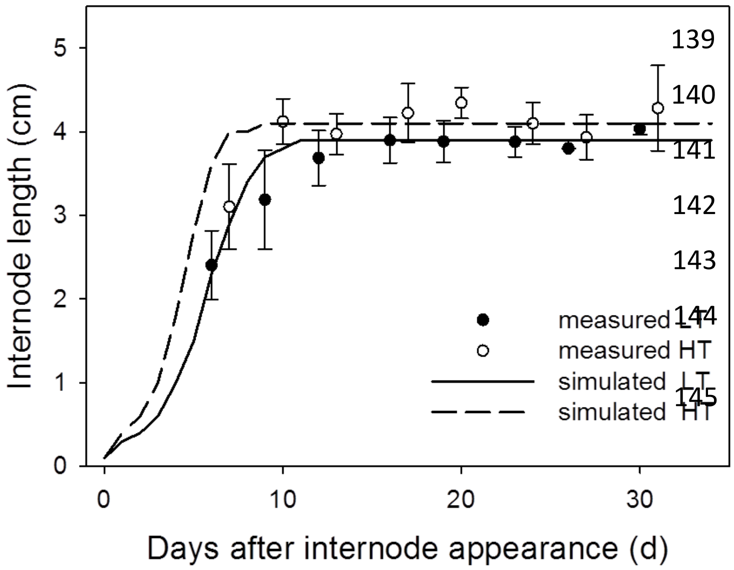

Fig. S7. Measured (symbols) and simulated (lines) internode lengths at rank 8 at 22/18°C (LT, closed circle) and 32/28°C (HT, open circle) day/night temperature conditions (Expt. 5, n = 4). Bars are standard errors.

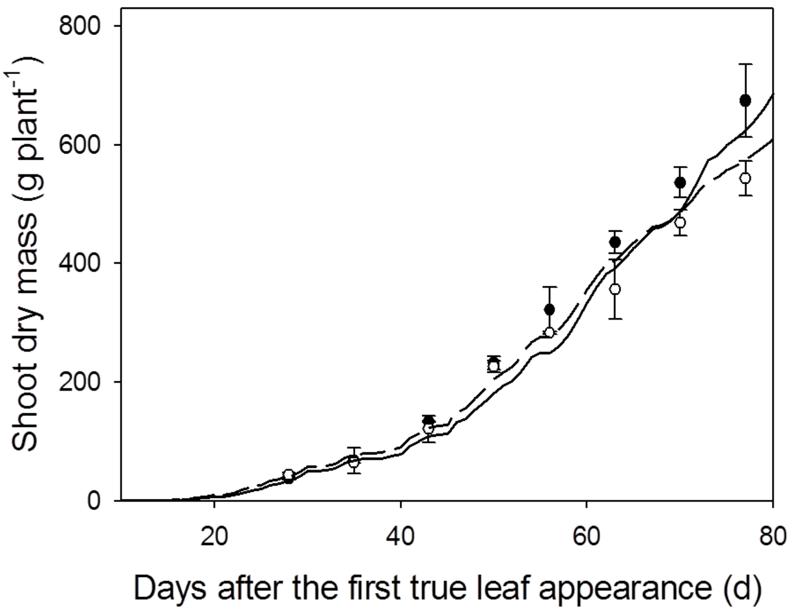

Fig. S8. Comparison between simulated and measured shoot dry mass at 22/18°C (LT, closed circle) and 32/28°C (HT, open circle) day/night temperature conditions (Expt. 5, n = 4). Bars are standard errors. Lines represent the averages of simulated shoot dry mass without temperature effect on light use efficiency (the term $(1-\kappa(T(t)-T^*)^2)$ in Eqn 12) under LT (solid line) and HT conditions. For LT condition, RMSD, bias and accuracy were 44.94 g, 38.84 g and 85%, respectively. For HT conditions, RMSD, bias and accuracy were 17.39 g, -1.55 g and 93%, respectively.

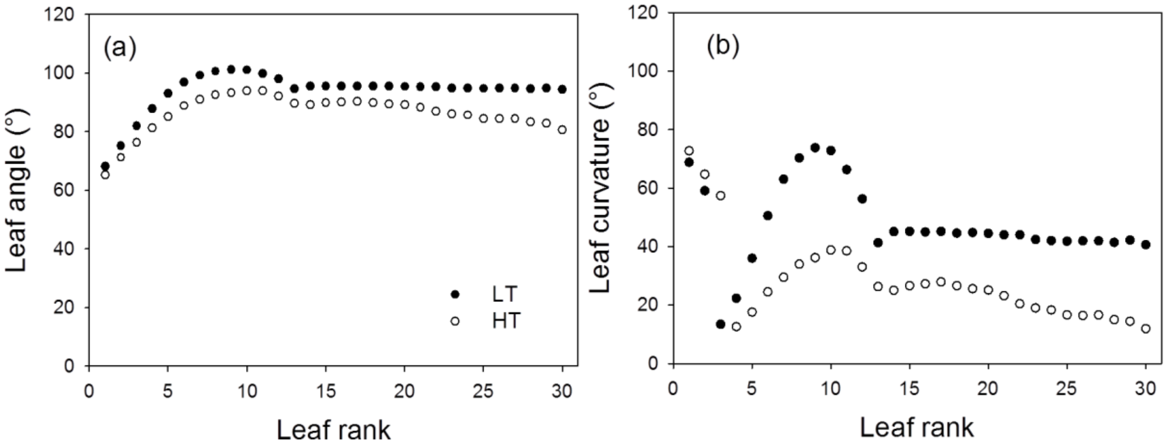

Fig. S9. Leaf angle (a) and leaf curvature (b) along the leaf rank on day 77 after appearance of the first true leaf at 22/18°C (LT, close circles) and 32/28°C (HT, open circles) day/night temperature conditions.
